# The Kids’ Environment and Health Cohort: a national, linked data resource for environmental child health research

**DOI:** 10.23889/ijpds.v8i3.2291

**Published:** 2023-09-11

**Authors:** Alister Baird, Emilie Courtin, Steven Cummins, Samantha Hajna, Vahé Nafilyan, Alison Macfarlane, Jess Walkeden, Pia Hardelid

**Affiliations:** 1 Population, Policy and Practice Research and Teaching Department UCL Great Ormond Street Institute of Child Health, University College London (UCL), London, UK; 2 London School of Economics and Political Science, London, UK; 3 London School of Hygiene and Tropical Medicine; 4 Brock University, St Catherine’s, Canada; 5 Office for National Statistics, Newport, UK; 6 City, University of London, London UK

## Introduction & Background

Evidence is mounting that children’s physical environment (e.g. in and around the home, school, and neighbourhood) is critical for their long-term health and education. Early life exposure to factors such as indoor and outdoor air pollution, or a lack of access to greenspaces are associated with the development of long-term health conditions such as asthma or mental health problems. Local and central government in England are implementing numerous policies to improve air quality and housing, and mitigate climate change. Further, England has seen large scale changes to local service provision (including childcare and libraries) due to austerity policies and the COVID-19 pandemic. Currently, there is no national, linked data resource for England that allows research into how the local environment impacts children’s health and education.

## Objectives & Approach

The Kids’ Environment and Health Cohort will be a new, linked national data resource for England currently being developing by researchers from UCL, London School of Hygiene and Tropical Medicine, London School of Economics and Political Science, Brock University, and City, University of London in collaboration with the Office for National Statistics (ONS), and funded by Administrative Data Research-UK (ADR-UK). The Kids’ Environment and Health Cohort will be a de-identified and annually updated national birth cohort of all children born in England from 2006 onwards – around 10.5 million children until 2023. The cohort will be constructed using linked administrative data from vital registration (live and stillbirth, and death registration), Census (housing and socio-economic indicators), health (hospital contacts, mental health referrals, and community dispensing data), and education (key stage results, special educational needs, absenteeism). Environmental exposure data can be securely linked to the Cohort via longitudinal residential unique property reference numbers (UPRNs) and postcodes from the Personal Demographic Service, and school location from education records.

## Relevance to Digital Footprints

The Kids’ Environment and Health Cohort will, for the first time, link health, education, Census and environmental data at national level in England. It will allow researchers to integrate data on local environments, including physical characteristics (such as temperature, building energy efficiency, or greenspace access) or the social environment (including proximity to food outlets, or services like libraries) with individual level data on health and education outcomes in children. This will be done using the ONS’s 5 safes framework, ensuring highest standards of data security and confidentiality.

## Results

The Kids’ Environment and Health Cohort will be constructed using administrative datasets, including national linked vital statistics, health, education and Census data from multiple data providers (ONS, NHS England and Department for Education), combined with small-area level environmental data for England. Together, these datasets allow detailed analyses of the impact of environmental exposures on health and education outcomes in children, with robust confounder adjustment. The Kids’ Environment and Health Cohort will be made available in a de-identified format in the ONS Secure Research Service (SRS).

## Conclusions & Implications

The Kids’ Environment and Health Cohort will provide researchers secure access to a national data resource integrating environmental and administrative health and education data, for child public health research.

